# Towards Parallel Selective Attention Using Psychophysiological States as the Basis for Functional Cognition

**DOI:** 10.3390/s22187002

**Published:** 2022-09-15

**Authors:** Asma Kanwal, Sagheer Abbas, Taher M. Ghazal, Allah Ditta, Hani Alquhayz, Muhammad Adnan Khan

**Affiliations:** 1 School of Computer Science, National College of Business Administration & Economics, Lahore 54000, Pakistan; 2 Department of Computer Science, GCU Lahore, Lahore 54000, Pakistan; 3 School of Information Technology, Skyline University College, University City Sharjah, Sharjah 1797, United Arab Emirates; 4 Center for Cyber Security, Faculty of Information Science and Technology, Universiti Kebangsaan Malaysia (UKM), Bangi 43600, Malaysia; 5 Department of Information Sciences, Division of Science and Technology, University of Education, Lahore 54000, Pakistan; 6 Department of Computer Science and Information, College of Science in Zulfi, Majmaah University, Al-Majmaah 11952, Saudi Arabia; 7 Department of Software, Gachon University, Seongnam 13120, Korea

**Keywords:** bottom-up attention, cognitive agent, parallel selective attention, psychophysiological states, top-down attention

## Abstract

Attention is a complex cognitive process with innate resource management and information selection capabilities for maintaining a certain level of functional awareness in socio-cognitive service agents. The human-machine society depends on creating illusionary believable behaviors. These behaviors include processing sensory information based on contextual adaptation and focusing on specific aspects. The cognitive processes based on selective attention help the agent to efficiently utilize its computational resources by scheduling its intellectual tasks, which are not limited to decision-making, goal planning, action selection, and execution of actions. This study reports ongoing work on developing a cognitive architectural framework, a Nature-inspired Humanoid Cognitive Computing Platform for Self-aware and Conscious Agents (NiHA). The NiHA comprises cognitive theories, frameworks, and applications within machine consciousness (MC) and artificial general intelligence (AGI). The paper is focused on top-down and bottom-up attention mechanisms for service agents as a step towards machine consciousness. This study evaluates the behavioral impact of psychophysical states on attention. The proposed agent attains almost 90% accuracy in attention generation. In social interaction, contextual-based working is important, and the agent attains 89% accuracy in its attention by adding and checking the effect of psychophysical states on parallel selective attention. The addition of the emotions to attention process produced more contextual-based responses.

## 1. Introduction

Parallel selective attention has recently become a significant conceptual motivation for multidisciplinary neuroscience and cognitive psychology research. These knowledge domains laid the foundation for designing powerful mental resource management and information-processing mechanisms for socio-cognitive agents [1]. Recent work has exposed the need for selective attention and information abstraction to structure, parse, and organize perceptual and sensory information for socio-cognitive agents that are going to interact with a real-time environment [2]. Attention is the process that is used to manage many perceptible activities, such as sensory integration, searching, alertness, interaction, memory management, decision-making, action selection, and execution [3,4].

Proficient selective attention in socio-cognitive agents is composed of bottom-up (stimulus-based) and top-down (goal-driven) attention cycles [5]. Top-down attention is a controlled process based on internal desires and intentions [6,7]. This process is initiated within working memory and creates the generation of desired requirements that move towards perceptual units of the system to select goal-oriented input [7]. Moreover, the bottom-up approach generates attention toward the stimulus’s essential and highly noticeable assets [8]. The study is grounded on spatial, temporal, and cross-modal attention mechanisms [9]. Spatial attention is a process that directs attention towards a location in space [10]. Temporal attention is employed to associate brain resources with incoming events on time [10]. Cognitive individuals work in a complex multi-stimulus environment, initiating coordination processes to generate attention across modalities. This collaborative work concerns cross-modal attention [8,11]. In any functionally cognitive agent, psychophysical states generate a vital role in the modulation and regulation of any attention process.

The psychophysiological states, such as emotions and motivations, are utilized to trigger each other to draw their combined effect on attention generation. Despite working parallel selective attention cycles, the functional competency of such socio-cognitive agents is questionable. In human cognition, physical conditions such as the motivation and emotion of hunger, thirst, sleep, and psychological states significantly affect the regularization of attention [12,13]. In any cognitive process, two types of emotional inhibition (endogenous and exogenous) fine-tune the attention selection mechanism to extract and prioritize the essential features from sensory information [14,15]. In any visioning process, emotionally linked targets obtain sustained and faster attentional searching than non-emotional targets [14,15,16]. Motivation has two types, intrinsic and extrinsic, which influence the consistency and searching of any attention process. In extrinsic motivation, defined incentives can continue a person’s attention towards a particular activity. In contrast, in intrinsically motivated action, the attention mechanism generates fewer mistakes compared with extrinsically motivated activity [17,18,19].

A socio-cognitive agent requires extracting the significant features from multimodal sensory information and combining them according to their semantically associated context under the influence of its psychophysiological states. Many cognitive architectures, such as Ymir [5], iCub [20], and QuBIC [21] are multisensory architectures whereas CHREST [22], NARS [23], LIDA [24], AKIRA [23], OPENCOG PRIME [25], CLARION [26], ACT-R [27], and AISA [28] mainly work with single sensory input. They perform overt or covert attention and use limited visual or auditory features. The limited use of features approach produces unexpected environmental responses. There is a need to understand if sensing a single feature is enough to generate a human-like attention process and what other features are essential for parallel selective attention. These architectures mostly perform spatial- or temporal-based selective attention and disregard the importance of cross-modal attention in multimodal agents. These architectures do not include psychophysiological states to regulate attention mechanisms. This insufficiency shows that these agents encounter difficulty generating effective and timely context-based attention processes in multi-modally sensed information. As the literature defines, psychophysical states generate a significant role in selective attention generation. So, it is essential to check what psychophysical conditions are necessary and how they influence each other.

Considering the limitations of existing architectures, this research aims to propose a computational cognitive model for socio-cognitive agents that incorporates psychophysical states to generate a human-like parallel selective attention process.

This work comprises on the following objectives:

1. Identifying the significant audio-visual features for parallel selective attention.
2. Performing a comprehensive analysis of existing systems that use a selective attention mechanism.
3. Analyzing the role of psychophysical states on parallel selective attention mechanism.
4. Designing of a human-inspired parallel selective attention mechanism for an artificial agent.
5. Testing and verifying the behavior of the proposed attention mechanism for cognitive architecture in a virtual or real environment.

The following is a breakdown of the paper’s structure: Section 2 comprises related work; Section 3 covers the proposed conceptual model and internal modules of the proposed work; Section 4 presents the results and discussion; Section 5 is a discussion of the conclusion; and Section 6 comprises the future work.

## 2. Related Work

The study defines that many cognitive architectures manage the attention process, but each has feature-based or computational limitations described later.

### 2.1. Quantum and Bioinspired Intelligent and Consciousness Architecture (QuBIC)

QuBIC [21] architecture comprises the concept of self-awareness and consciousness to achieve general intelligence. This architecture consists of many cognitive phenomena on different layers to achieve consciousness at a certain level. The architecture comprises the physical layer based on sensors and actuators. Then it has an unconscious layer for the self-regulation of the artificial agents. The conscious layer is responsible for the intention, attention, awareness, and handling of voluntary tasks. This architecture performs conscious behaviors based on different memories, such as sensory memory, to get input from the environment. Perceptual associative memory formulates the association between the perceived intakes. It has a decision executor center for perception and decision of the overall system. This module consists of short-term memory, working memory, and long-term memory. Then it has a circadian clock for monitoring and controlling. The seed knowledge module in it comprises a predefined knowledge base. Then the deed assessment module assesses the decisions generated by the system. Other modules such as dream and imagination are used to design dreams for the system. Then the imagination module generates creativity based on some story-based input. Then it has an emotion module to extract emotions from facial expressions. The meta-cognition module controls the overall system’s conscious and unconscious processing. This architecture will analyze the quality of its consciousness under the scales defined by consScale.

### 2.2. LIDA

The LIDA [24] architecture is an intelligent and autonomous software agent. This architecture is an autonomous US Navy negotiating software responsible for automatically generating assignments for personnel according to the US Navy policies, sailors’ preferences, and many other factors. This architecture is based on the global workspace theory of consciousness [14]. The LIDA memory system consists of sensory-motor, perceptual, episodic, declarative, and procedural memory. LIDA architecture performs in multiple cognitive cycles, each composed of numerous mental processes and sense, attention, and action selection phases. The attentional codelets are responsible for transferring this functionally associated information to the global workspace from the local workspace. The global workspace, based on urgency value of incoming information, broadcasts it into the whole system. Many architecture processing components receive this information for learning, memory, and decision-making purposes. In LIDA architecture, attention is implemented for selection, generating the system’s capability to prevent information overloading. The attentional learning in LIDA allows it to perform resource management in terms of data filtering intelligently. The primary learning mechanism of the architecture is fixed, but internal data management is performed according to external data. The LIDA architecture performs introspection and self-improvement at the content level.

### 2.3. Vector LIDA

Vector LIDA [29] is the variation of LIDA architecture. This architecture has modular composition representation (MCR) for its representation module. It uses integrated sparse distributed memory for the primary memory implementation. The previous LIDA version used a graph-based structural approach for generating multiple memories. Previous LIDA architecture has approximate computation in its graph-based structuring. Secondly, some modules in LIDA require learning-based mechanisms, such as perceptual associative memory, episodic memory, and procedural memory. Vector LIDA applies a reinforcement learning mechanism in its memories. The extension in the current work makes the overall representation of the memories more realistic and biologically plausible.

Considerable work has been performed to introduce an attention mechanism in computational and cognitive models. Table 1 presents a detailed analysis of previously performed adaptive working in agents.

### 2.4. Limitations of Existing Cognitive Architectures

Attention generation and regulation are limited in the scope of working memory. Some work previously performed on adaptive working memory defines attention-oriented qualities [30]. QuBIC is artificial general intelligence (AGI)-based, quantum, conscious framework designed to work with audio-visual modalities [21]. This model is limited to some features and does not exhibit the influence of psychophysiological states on bottom-up and top-down attention processes. OpenCog PRIME is a cognitive architecture [25] envisioned for implementing artificially intelligent systems capable of holding common knowledge representation. OCP addresses only attentional knowledge, aiming to focus on what information should obtain resources at every moment. The LIDA architecture [9,24] is planned to design an autonomous and artificially intelligent software agent. LIDA implements attentional learning and provides an architecture to improve internal resource management by filtering data. The primary learning mechanisms in LIDA architecture are non-evolutionary. In the LIDA architecture, internal data are handled identically to external data.

iCub is an artificially intelligent humanoid robot with a multimodal approach; it has many cognitive abilities, such as gazing and grasping. iCub performs a bottom-up attention mechanism using audio-visual features [20]. The limitation of its attention mechanism is that it works with limited audio-visual features and performs predefined, precise, region and situation-based attention. Although they work with multimodal attention, they do not achieve cross-modal attention focus [8]. Therefore, their work is limited to the bottom-up approach of attention. Their working system could not work with targeted attention to intelligently utilize internal resources.

## 3. Materials and Methods

### 3.1. Parallel Selective Attention (PSA) Model

The human attention process is controlled by many psychophysical states to change/shift the attention process based on demanding goals. Human-like, effective attention processing demands multimodal and multi-feature-based attention. The primary focus of this research is to introduce the parallel selective attention mechanism in functional cognitive agents by designing a conceptual, computational cognitive model.

**Figure 1:**
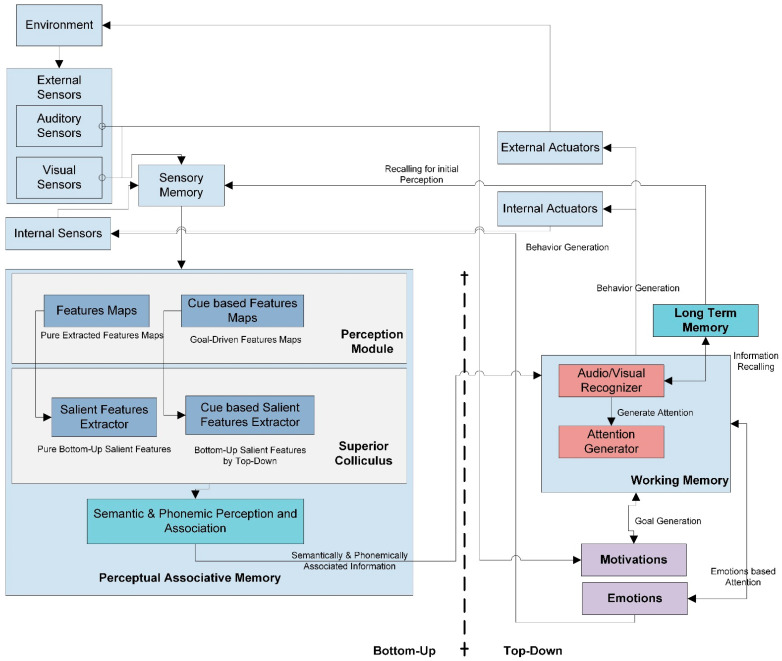
Parallel selective attention (PSA) model. This model represents the overall working of the bottom-up and top-down attention processes. This model also defines the link of various memories for generation and regulates the attention process.

This work proposes a cognitive agent that is based on cognitive neuroscience. Cognitive neuroscience’s main objective is comprehending how basic brain functions, human thought, and behavior are related. The field focuses on the mechanics of the brain and the localization of brain activity in response to human actions, ideas, and behaviors by using functional neuroimaging techniques. The tools provided by neuroscience and neurophysiological understanding can be used to comprehend better the development, use, and effect of information and communication technology (ICT). The development of novel hypotheses that enable accurate predictions of social cognitive-related behaviors, as well as the development of cognitive systems that positively impact economic and non-economic variables, such as productivity, satisfaction, adoption, and well-being, are made possible by artificial cognitive systems based on neuroscience. A few examples of the practical applications of cognitive neuroscience include technology adoption, virtual reality, technology stress, website design, virtual worlds, human-computer interface, social networks, information behavior, trust, usability, multitasking, memory, and attention [31,32,33]. In light of the field of neuroscience, PSA created an attention strategy for an artificial agent employing artificial components with a neuro-cognitive base. The model generates attention to the most salient features and objects of the sensed audio-visual information by considering their internal and external psychophysiological states [34]. For the selective attention process, PSA uses Treisman’s attentional model as the core model for the generation of attention processes for visual attention [35]. The auditory attention process uses cocktail party phenomena as the primary model for acoustic attention generation [36]. PSA (Figure 1) performs its tasks using many interconnected cognitive subsystems: perceptual associative Memory (PAM), working memory (WM), and long-term memory (LTM).

#### 3.1.1. Sensors

This model consists of internal sensors that sense the agent’s internal motivational and emotional states and two external sensors, audio and visual. These sensors pass their inputs to the sensory buffer, temporarily saving input for further processing.

This buffered information passes to perceptual associative memory, further divided into two submodules: perceptual module and superior colliculus.

In each stage of the intelligence process, two types of selective attention mechanisms work in parallel: bottom-up attention and top-down attention. Therefore, the perceptual module has two submodules to initiate the phenomena of bottom-up and top-down attention.

#### 3.1.2. Top-Down and Bottom-Up Attention

The bottom-up approach performs simple, audio-visual, low-level feature extraction.

##### Perceptual Module

The perceptual module input image will pass through a convolutional neural network layer known as the feature network (FN) [37]. The output of this FN consists of convolutional feature maps. The learned filters produce multiple channels of features. These purely extracted features then pass to the normalization phase. In the normalization phase, this model performs two suboperations. First, the contrast between two extracted features using the center-surround difference is determined. The model will normalize the feature value in the fixed range in the second step. In audio feature extraction, the Mel frequency cepstral coefficient (MFCC) is applied to extract the frequency and loudness features [38,39]. Just like visual input, these extracted features are then normalized and the center-surround difference will be calculated between these features. This study uses the means and variance normalization (MVN) technique for audio feature normalization [40,41]. These generated feature maps will transfer to the superior colliculus.

##### Superior Colliculus

The superior colliculus consists of the regional proposal network (RPN) [42]. First, the RPN produces conspicuity maps for each audio-visual feature by combining their feature maps. Second, the model performs normalization on each conspicuity map. Third, the model will combine all conspicuity maps to generate a single convolutional saliency map. This saliency map attains the most salient audio-visual features of all feature maps. Therefore, for top-down attention, region of interest (ROI) pooling is performed in a new tensor matrix [42].

##### Perceptual Associative Memory

These bottom-up-based and top-down-based saliency maps then shift to PAM, drawing generic semantic and phonemic associations between two features in bottom-up attention. Top-down attention marks attention-based, semantic priming between two visual features [43]; additionally, it relates to phonemic priming for auditory features. PAM is accountable for performing the semantic, structural, and phonemic analysis of input signals and their correlation. These semantically and phonemically associated features are then sent to working memory. The Dirichlet mixture model (DMM) [44,45] is used to abstract cue-based, auditory semantics, and the Markov chain Monte Carlo (MCMC) [46,47] technique is used to extract visual semantics. The hidden Markov model (HMM) [48,49] technique is preferably employed to generate an association among audio, visual, and auditory-visual features. Phonemic perception of the auditory signal is achieved through the TRACE method [50].

##### Working Memory

Working memory [30,51] has two submodules to process incoming information.

1. Audio-Visual Recognizer:

An audio-visual recognizer, known as a dense network, is designed with a recurrent neural network (RNN) and is responsible for the final classification [52,53]. This network recognizes the targeted visual and auditory inputs. This module performs the recognition process by recalling past information from long-term memory. Long-term memory holds previously learned implicit and explicit knowledge and past agent experiences about current auditory and visual input. The audio-visual recognizer sends its response to the attention generator.

2. Attention Generator:

An attention generator based on preprocessed signals from audio-visual recognizers and identified locations from ROIs finalizes the locations where attention needs to be generated. The attention generator will decide on the suitable action to produce attention as a response to the environment. The attention generator will send these definite actions to an external actuator to generate attention-based behavior in the environment.

##### Long-Term Memory

Long-term memory is considered the knowledge base, consisting of semantic [43] and phonetic memory and context-based emotion and motivation rules designed with fuzzy logic.

##### Psychophysical States

In PSA, internal psychophysiological states such as motivation assist working memory for goal generation. Emotion directly links working memory to produce attention by considering associated internal emotional states [54]. External audio-visual input signals also generate their influence on internal motivational and emotional states. In the implementation aspect, the model’s current development uses the James-Lange theory of emotions to associate specific emotions with objects and broaden and build a theory used for promoting actions according to positive and negative emotions [55,56,57].

##### External and Internal Actuators

In PSA, internal and external actuators are considered output units. In PSA for external actuators, we consider object detection and recognition as the final response to the environment. Whereas for an internal response, the internal sensors are linked with PSA’s emotions and motivation states. These psychophysical states are considered, and internal actuators and internal responses are used as a state in regulating emotion and motivation states for the next incoming input.

The PSA model has some computational limitations like other cognitive systems, for example, humans can compute attention processes by considering all physical features in bottom-up attention. In this research, we are considering only five physical features. All computational systems have limited memory for the storage of learning, and they provide problem-based solutions. The same limitations exist with PSA. The PSA model does not provide an artificial general intelligence-based generic solution. Artificial neural network-based systems also have limitations in learning computational complexity. The same problem exists with PSA. For the current implementation, PSA uses only emotional features to analyze the overall working system. Still, humans can generate attention by considering many other psychophysical states. Even in humans, internal primary and secondary drives also play significant roles in generating and regulating attention. Still, for current artificial agents, these drives are not accommodating.

### 3.2. Functional Flow of PSA

In this section we explain the function flow of PSA. Table 2 represents the all used notations of internal and external states which used in flow of PSA. Similarly, Table 3 represents the overall functional working of flow of PSA along its description.

### 3.3. Dataset

The model can classify data into 80 different classes. The dataset selected for training and testing the model was Coco-2017 [58]. To train, test, and validate the classifier, 118,287 samples, 40,670 samples, and 5000 samples were employed.

### 3.4. Experiment Setup

PSA is currently partially implemented for parallel selective visual attention in a socio-cognitive agent using color and motion features. The developed agent can manage and prioritize the upcoming information according to the predefined context. This prioritization makes independent decisions to generate proper reasoning for incoming signals.

#### 3.4.1. Bottom-Up and Top-Down Attention Cycle of PSA

This experimental setup is designed to assess the initial working of the proposed model to analyze the bottom-up and top-down attention cycles without the influence of the emotional state on it. These experiments are currently based on single-object recognition. In the bottom-up cycle, the agent sensed the live stream of video through its visual sensor and sent it to sensory memory. Sensory memory buffered these frames and transferred them to PAM for perception. In any real environment, salient colored features and motion in the scenes are attended by the viewer through bottom-up attention [59]. Considering this idea, PAM extracts low-level, salient feature extraction, such as color and motion, and performs face and object recognition processes. PAM calculates the potential deficit of received information using (1).

(1) $$P.D=AS_{PAM}\times W_{PAM,STM}$$

PAM performs blob detection on all salient colored and moving objects or faces in the sensed scene. These salient features shift towards short-term memory (STM), which is responsible for passing it to WM for further processing. STM will calculate the activation value of the sensed signal with the following given equation

(2) $$AS_{STM}=1-P.D$$

##### Scenario

**Figure 3:**
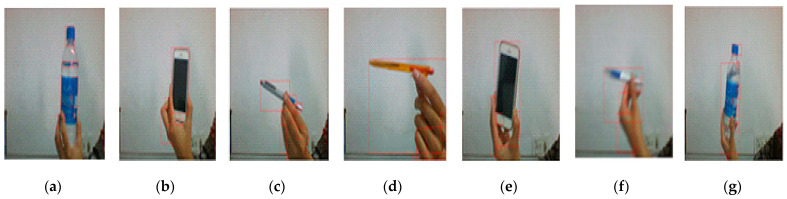
Scenario performance of PSA for both bottom-up cycle and top-down cycle. This figure defines the recognition of different objects through PSA bottom-up and top-down attention processes. (**a**) Define the bottle object with significant bright color detection. (**b**) Is the recognition of moving cell phone with orientation change, which can miss the actual recognition of object. (**c**) Define a pen with orientation change, (**d**) show the pen in moving condition. (**e**) Shows the cell phone in straight condition for clear vision of object. (**f**,**g**) shows pen and bottle respectively with fast moving rate to find the performance of agent for fast object recognition.

A scenario is designed to evaluate the interplay between the bottom-up and top-down cycles of selective attention. To address this interplay, another experiment is performed by applying the selective attention approach to different objects. In bottom-up attention, this agent will start obtaining input from sensory memory, and PAM will perform initial perception based on low-level features. PAM initial perception will extract the blue-color object, brown strip, and hand in this cycle. This experiment also performs the top-down approach of selective attention to focus on a particular thing. In this experiment, WM designs a predefined goal for attention generation. In the top-down cycle of selective attention, WM decides in advance to concentrate on the blue-color bottle object. WM retrieves relevant details about the goal from LTM, designs the necessary attentional parameters for PAM, and then passes the attentional parameters to PAM. PAM will search for suitable objects in the incoming scene through the object recognition process and give this information to the STM through the goal-based searching mechanism. The STM calculates the activation value of the signal and transfers these relevant details to the focus of attention (FoA). This module will apply the attention regulation process to switch agents’ attention to the defined blue-color bottle. Multiple experiments are performed by changing the goal in WM. Figure 3 shows the agent performance in the top-down selective attention process.

The resultant Table 4 consist of multiple parameters, and frame per second (FPS) defines the number of frames per second ratio. None of the objects defined in each scenario represents the number of blobs created on each object. The motion level determines the object movement level in a scene, and three parameters specify each scene’s red-, green-, and blue-color ratios. Motion and color are low-level features that are identified in each frame. ASpam shows the filtered signal of object and face recognition performed during top-down attention. In contrast, this parameter represents the filtered signal for motion and color detection in the bottom-up attention case.

There are many other features, such as Wpam showing the activation signal for working memory to PAM during the top-down approach; potential deficit (PD) is the potential deficit value for each frame. ASstm represents the activation signal for STM. New potential deficit means a new potential deficit for each frame. These results define two scenarios in which a socio-cognitive agent for attention generation performs. FPS defines the ratio of the total number of frames per second to start the bottom-up attention process. If the FPS ratio increases, it helps the agent identify objects in each case in a better way. These graphs define the relationship between the dependent cognitive states of the agent, as it calculates the motion level and colors (red, green, blue) in each frame. If the value of the motion level and colors is high, the agent will generate stronger activation signals (ASpam) towards perceptual associative memory for bottom-up attention generation. If an agent obtains a higher ASpam value, these strong signals activate the weight between PAM and STM, labeled Wpam. Based on ASpam and Wpam, STM calculates the potential deficit in the incoming signal. STM also calculates ASstm, which is the motion deficit for STM. The calculation of these new potential motion deficits was estimated to generate the top-down selective attention process.

**Figure 4:**
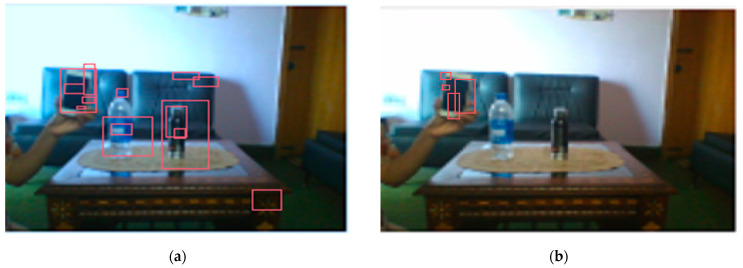
(**a**) PSA initial performance in a multicycle environment (bottom-up attention). This experiment defines that in a bottom-up attention process, PSA system will generate attention on every prominent feature of the object. (**b**) PSA initial performance in a multicycle environment (top-down selective attention). This experiment defines the PSA attention process on only the targeted objects. Here, PSA is asked to pay attention only to mobile objects. So, PSA recognizes the object that was asked for as a priority and pays its full attention to the mobile object.

Variations were performed in these experiments to observe the performance of PSA in a multicycle environment to observe and recognize multiple objects in the scene. Working memory decides that a goal for an agent is to focus on a mobile phone. During its bottom-up attention, the PSA agent focuses on all the salient objects it visualizes in the environment, as defined in Figure 4a. Figure 4b defines the performance of PSA in the top-down selective attention process.

## 4. Results and Discussion

### 4.1. Comparative Experiment

Initially, for the attention process, training of PSA was performed on the Coco 2017 [58] dataset with 20 classes. Table 5 elaborates on the number of classes and instances.

This working system is currently used only for 20 classes of objects to check the regulation and effect of emotions on context-based selective attention. The results analyze the working of object recognition with and without an attention process. The implementation of this work will determine the extent to which emotions help attention regulate and achieve goals according to the defined context.

**Figure 5:**
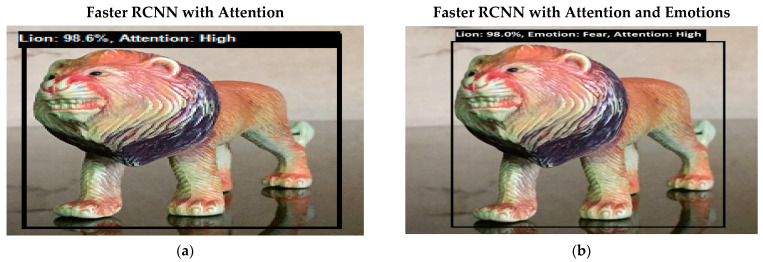
(**a**) Bottom-up attention-based object recognition without internal emotions. Now the system is fully focused on the lion object. (**b**) Bottom-up attention-based object recognition with the system’s internal emotions. Initially, the emotions of the system were normal, but as the system recognizes a given object, its emotions change to fear. Fear is a strong emotional state, so it regulates a medium level of attention to a high level.

**Figure 6:**
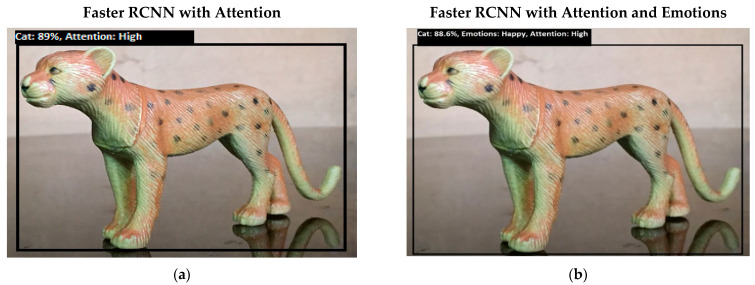
(**a**) This figure shows the bottom-up (feature-based) attention-based recognition of an object such as a cat with 89% accuracy, and the attention level is high. (**b**) This figure shows the bottom-up (feature-based) attention-based recognition of an object such as a cat, and the emotion of the system converts to a happy state from the previous fear emotion. Happy is an intense emotional state, so attention is high.

**Figure 7:**
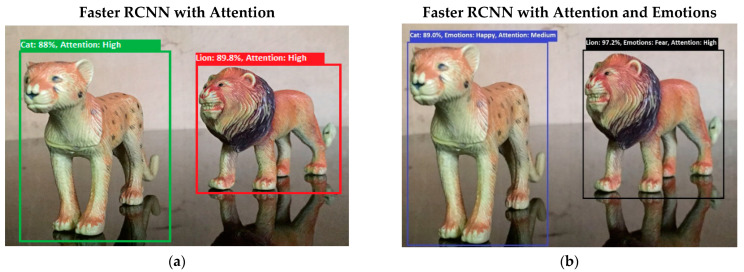
(**a**) This figure shows the recognition of lion and cat objects. Results show that both objects have high attention levels in the bottom-up attention process. (**b**) This figure shows the recognition of lion and cat objects, but the emotions associated with both objects are different. Both emotions fall in a strong emotional state category, but fear emotion has higher intensity, so attention toward a lion is more intensive than it is toward a cat. Figure 7 shows the bottom-up attention regulation under the influence of internal emotions.

**Figure 8:**
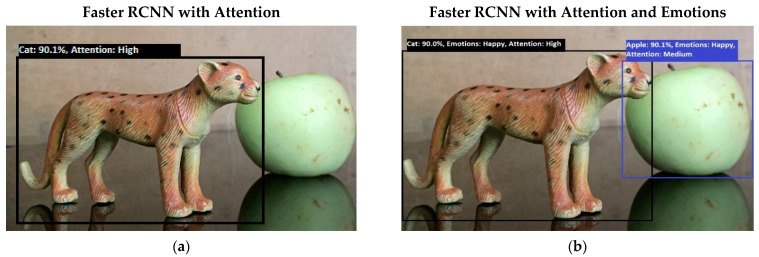
(**a**) The system’s performance in the top-down (goal-driven attention) attention process. The system generated a query to “find the cat” in the given picture. The intelligent system recognizes cat objects with a high attention level. (**b**) This figure shows the system’s performance in the top-down (goal-driven attention) attention process. The intelligent system recognizes both objects: apple and cat. Both objects have happy emotions, but the attention of the system diverts towards the target object. The system generated a query to “find the cat” in the given picture.

**Figure 9:**
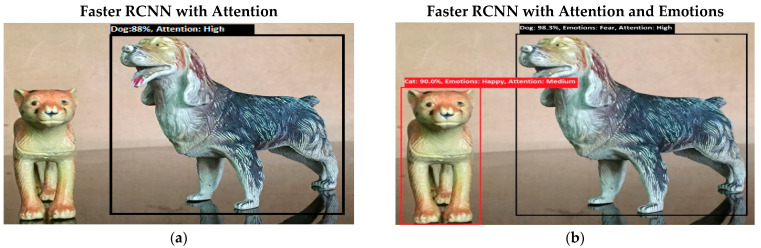
(**a**) The performance of the system against the query to “find Dog”. Attention is regulated towards the dog object under the influence of asked query. (**b**) The performance of the system against the query to “find Dog”. In this experimental PSA system, internal emotions shift towards fear from happy emotions. Attention is regulated towards the dog object under the influence of asked queries and emotions.

**Figure 10:**
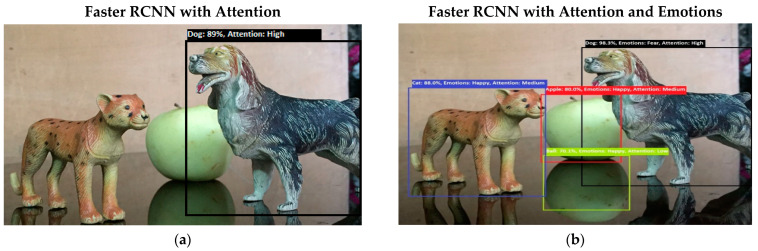
In (**a**), the same query as the previous one was generated to detect dog objects under the working of top-down attention. The system consolidates its attention toward the queried object. In (**b**) experiment, the same query as the previous one was generated to detect dog objects under the working of top-down attention. The system consolidates its attention toward the queried object based on strong fear emotion. In the following experimental results, some misrecognition occurs with the apple reflection. The system considers the apple reflection a ball object.

**Figure 11:**
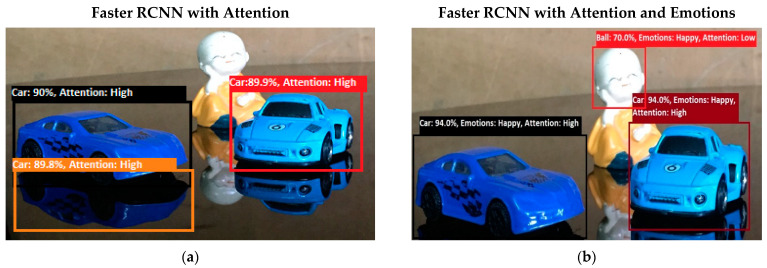
(**a**) In this experiment, the artificial system was queried to “find a car” based on the top-down attention process. This experiment is an example of multiple-object attention. The system recognizes the objects asked for with a high attention level. The following experiment system wrongly detects the car’s reflection as a real car object. In test experiment 7, shown in (**b**), the system was queried to “find a car” based on the top-down attention process. This experiment is an example of multiple-object attention. The system recognizes the object asked for with a high attention level. In the following experiment, the system wrongly detects the human face as a ball. This wrong detection occurs due to messed-up features of humans, which are not properly identified by the system.

**Figure 12:**
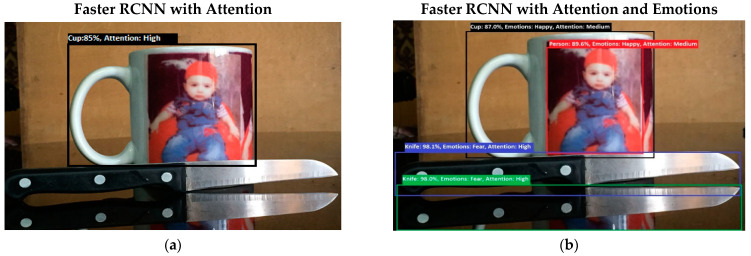
(**a**) In this experimental result, PSA system performs multiple attention processes. The system finds the desired object with a high attention level by ignoring other objects. In the given experiment, PSA was queried to “find Cup”. (**b**) Results from experiment 8 show PSA system performance in multiple attention processes. In the given experiment (**b**), PSA was queried to “find Cup”. The system finds the desired object, but the scene has other intensified emotional objects, so attention diverts toward those objects. Secondly, in the picture, as the knife reflection was clear in the mirror, the system wrongly counts the knife reflection as a knife.

**Figure 13:**
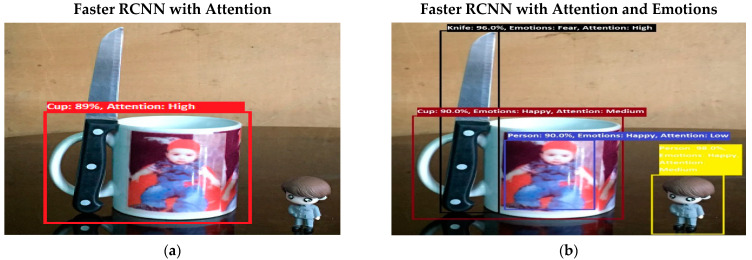
(**a**) In an experiment, the same “Find Cup” query was asked of the system again. Now system finds a cup with more precise recognition. In experiment 9 (**b**), the same “Find Cup” query was asked of the system again. Now system finds a cup with more precise recognition, but still the fear emotion forces the system to generate more attention toward the knife.

Software is generated by applying different machine learning techniques under the defined working flow of PSA. Then to assess the working of PSA, different situations are designed for PSA. Some results associated with the real-time performance of this work are discussed in the following section. As in human psychology, emotional solid objects get more attention than normal or low emotions. The following experimental results in Figure 5, Figure 6, Figure 7, Figure 8, Figure 9, Figure 10, Figure 11, Figure 12 and Figure 13, show the comparison between agent Faster RCNN recognition with attention only and, on the second side, agent recognition with attention and emotions.

The defined few experiments explain the diversion of attention towards the non-targeted object under the influence of active emotions. In our real-time working system, sometimes humans have predefined goal-based working, but runtime associates emotion with other non-targeted objects, and emotional intensity forces humans to divert from their target. In some weird situations, these emotional regulations are helpful to divert the sub-target to achieve the final goal. For example, in Figure 13b, if the target action is to “recognize and grasp the cup”, then only paying attention to the cup and ignorance of the knife will harm the grasping hand. In this situation, firstly, paying attention to the knife and carefully removing it from the position will increase the success and safe achievement of the targeted goal.

A summary of the above-discussed comparative experiments is given in Table 6. Table 6 defines the attention status and goal achievement without emotions.

Table 7 defines how emotions sometimes deviate attention towards non-goal objects just on the coming of highly emotional input. Table 7 displays the agent’s performance in various experiments when emotions are incorporated into the process of paying attention. When the goal is unclear in tests one through three, the agent pays close attention to objects having a high emotional valence (either positive or negative). When emotions are happy, as in tests four and seven, the agent is completely focused on the intended object, even though many things have pleasant feelings linked with them. In tests five and six, where emotions are negative (fear), the agent still concentrates on the goal object because these objects have a high level of negative emotion at that moment. In trials eight and nine, agents actively divert their attention from the goal object to non-goal objects because these non-goal things have negative emotions associated with them.

**Figure 14:**
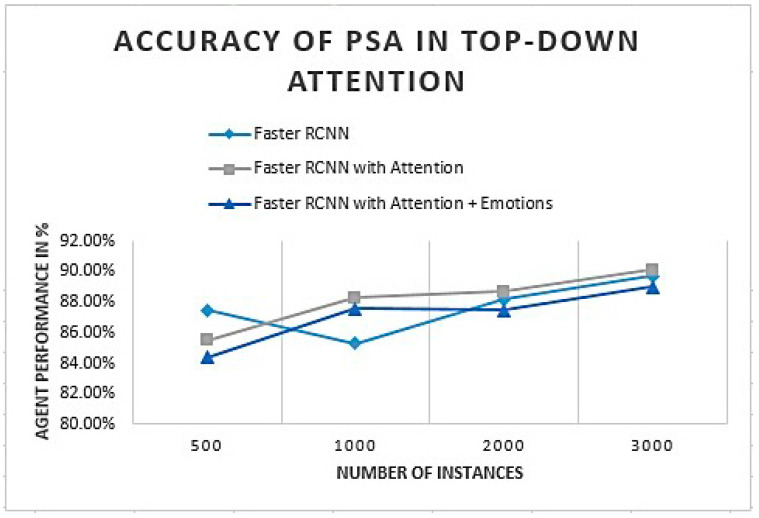
PSA performance given as a percentage in the top-down attention process.

Faster RCNN (recurrent convolutional neural network) performance was analyzed with and without an attention process. Analysis was performed on Faster RCNN with attention and emotions. The accuracy of PSA for given classes is defined in Figure 14.

The initial analysis of PSA defines an object recognition system that slightly improves by adding the attention process. Figure 14 shows that PSA accuracy improves as we attach an attention process to generate the focus toward the goal-based object. Attention to emotions slightly decreases the attention process as emotions deviate according to the incoming object and subjective experience.

### 4.2. Discussion

Attention is a critical human cognitive capability for surviving in a dynamic and complex environment. This ability allows a person to concentrate on the most relevant sensed information while reacting to unexpected events. Without this cognitive ability, alertness to frequent environmental changes while executing single/multiple important tasks is impossible [5]. Stan Franklin implants attentional learning in LIDA; it performs learning based on predefined contents [9]. It cannot change its attention according to situational changes in the environment. QuBIC [21] also copes with the attention mechanism in its framework, but multimodal spatial attention is performed by considering the limited features of audio-visual sensors [4]. iCub is also a well-known robotic environment that unites the attention mechanism; however, attention is designed for iCub only and based on limited audio-visual features [20,60]. Many other cognitive architectures and robotic environments generate attention mechanisms but limit their attention process. Some are based on a single modal, and some work with multimodal features but generate attention based on limited features. Some cognitive architectures and robotic environments work on predefined content-based attention, and their work is also limited to spatial and temporal attention [61].

Considering the aforementioned findings, a conceptual model is required to develop a parallel selective attention process for any cognitive agent based on psychophysiological states. In functional cognitive systems, during multimodal sensory information agents, internal psychophysiological states continuously guide them to generate attention on internally associative and important information sensing from the external environment. PSA can serve as a basis to sense the required information from the external environment and generate the best possible action as a result. PSA continuously senses audio-visual input from the external environment and emotions and motivations from internal sensors. PSA can semantically and phonetically associate audio-visual input with a generating attention mechanism. The internal controlling process of PSA continuously monitors it to generate attention on the most relevant chunks of information sensed from the environment. The internal psychophysiological states of PSA influence the attention mechanism. Integrating such cognitive states in a unified way is the basis for the cognitive machines, and PSA is a small contribution to this course.

## 5. Conclusions

Socio-cognitive agents struggle to develop a parallel selective attention mechanism that is human-like. Important factors such as multisensory input, cross-modal attention, or the impact of psychophysical states on attention control are frequently overlooked in contemporary architecture designs. A basic computational model for a parallel selective attention mechanism is presented in this paper by integrating bottom-up and top-down attention processing. PSA can develop an attention process by considering and integrating sensory data from both internal and external sensors. PSA performs real-time memory consolidation, signal analysis, and responsible production. To achieve goal-driven attention, this study investigates how emotional states and motivations impact the psychophysiological internal regulatory mechanism that controls attention. The results of the experiments demonstrate that the system recognizes the object more precisely when it is only using the attention process, but it ignores other significant factors in order to safely carry out its actions and sub-actions. Unexpected incidents can require more attention when working in real-time and put a heavy emotional strain on the agent. Finding a solution is of utmost importance because these highly emotional incidents call for urgent care. In its current implementation, PSA only evaluates the system’s emotional components to determine how it is performing overall. Individuals can draw attention, though, by taking into consideration a number of extra psychophysical states. The creation and control of attention in humans are also greatly influenced by internal primary and secondary impulses. The PSA model is still incompatible with these drives. 

## 6. Future Work

There are numerous ways to expand on this work. In the initial phase of extension, it will be practically tested to see what effect motivational states have on the control of the attention process. Alderfer’s ERG theory will be used in the implementation of the PSA model in the future [62]. Examining the impact of motivational states on the modulation of emotional states is the second extension of this research. Patterning the combined impact of motivations and emotions on attention management is another component of this approach.

## Figures and Tables

**Figure 2:**
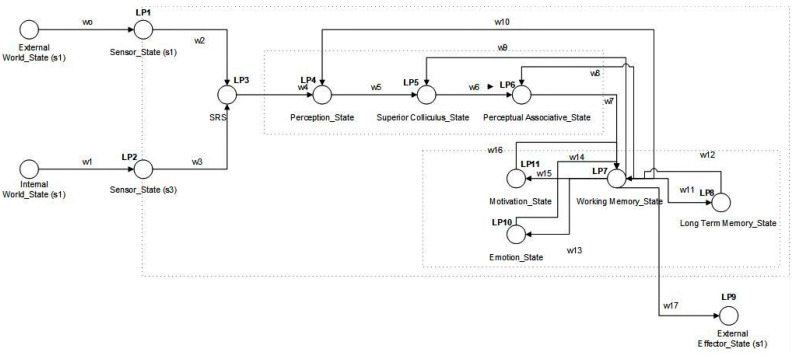
Functional flow of PSA. This figure defines the PSA model’s working flow by defining the different states of PSA in the light of formal methods. This figure also represents state switching of PSA on different conditional stages.

**Table 1:** Comparative analysis of different existing cognitive architectures.

Cognitive Architecture	Sensor Type		Attention Cycle Type		Attention Type			Attention Features			Memories
	Unimodal	Multimodal	Top-Down	Bottom-Up	Feature-Based	Object-Based	Other	Significant Feature	Predefined Feature	Other	Cognitive Memories
**iCub** [20]	No	Yes	No	Yes	Yes	Yes	Spatial	Yes (Ordinary)	No	Object detection	Yes
**AKIRA** [23]	Yes	No	Yes	No	Yes	No	Spatial	No	Yes	No	No
**Ymir** [5]	No	Yes	Yes	Yes	Yes	No	Spatial	Yes (Ordinary)	No	No	Yes, in frames form
**OPENCOG PRIME** [25]	Yes	No	Yes	No	No	Yes	Spatial			Economic attention network (attentional knowledge)	Yes
**CHREST** [22]	Yes	No	No	Yes	Yes		Spatial	Yes (Ordinary)	No	No	Yes, in schematic and chunks form
**QuBIC_Johi** [4,21]	No	Yes	Yes	No	Yes	Yes	No	No	Yes	Visionary and auditory	Yes
**LIDA** [24]	Yes	No	Yes	Yes	Yes	No	Temporal	Yes	No	Textual information	Yes
**Vector LIDA** [29]	Yes	No	Yes	Yes	Yes	No	Temporal	Yes	No	Content-based working	Yes
**Proposed Methodology**	No	Yes	Yes	Yes	Yes	Yes	Yes	Yes	No	Context-based object recognition	Yes

**Table 2:** This table defines the internal and external states of Figure 2.

Notation	Description
EWS(s)	External world state for s
IWS	Internal world state
SS (s)	Sensor state for s
SRS	Sensory representation state
PS	Perception state
SCS	Superior colliculus state
PAS	Perceptual associative memory state
WMS	Working memory state
LTMS	Long-term memory state
MS	Motivation state
ES	Emotion state
EES	External effector state

**Table 3:** This table defines the overall working of different internal and external states of Figure 2.

From State	To State	Weights	Process	LP
EWS(s)	SS (s1)	w0	Sensing external world state	LP1
IWS	SS (s3)	w1	Sensing internal world state	LP2
SS (s1), SS (s3)	SRS	w2, w3	Sensory memory representation	LP3
SRS/WMS	PS	w4/w10	Perception (feature extraction/goal-driven feature regularization)	LP4
PS/WMS	SCS	w5/w9	Perception refinement/salient feature assessment	LP5
SCS/WMS	PAS	w6/w8	Semantic top-down based association	LP6
PAS/LTMS/ES/MS	WMS	w7/w12/w14/w16	Preparing action (visual recognition and attention generation)/information recalling/emotion-based attention regularization/goal-based decision generation	LP7
WMS	LTMS	w11	Information storage	LP8
WMS	EES	w17	Action execution to the external world	LP9
WMS	MS	w15	Goal generation	LP11
WMS	ES	w13	Emotion-based attention	LP10

**Table 4:** This table defines the agent’s performance in the given scenario, including the features and how the agent performs in response to these features.

Features	Computed Features for Figure 3a	Computed Features for Figure 3b	Computed Features for Figure 3c	Computed Features for Figure 3d	Computed Features for Figure 3e	Computed Features for Figure 3f	Computed Features for Figure 3g
**Frame Per Second**	16.0667	16.0667	16	16	16	16	15.9333
**No. of Objects**	9	7	5	3	4	3	14
**Motion Level**	0.04341	0.09435	0.14763	0.12900	0.05761	0.09788	0.06630
**Red**	0.16079	0.18431	0.17647	0.17647	0.18431	0.18431	0.21961
**Green**	0.10196	0.11765	0.12156	0.10196	0.10980	0.10588	0.10980
**Blue**	0.04706	0.06667	0.08235	0.05098	0.05882	0.07451	0.10961
**ASpam (Activation Signals for PAM)**	1.22234	1.32193	1.23095	1.16507	1.33456	1.42375	2.89953
**Wpam (Weight for PAM)**	0.03831	0.02949	0.0364	0.03012	0.01656	0.01489	0.0
**Potential Deficit**	0.04683	0.03893	0.04484	0.03509	0.0221	0.0212	0.0
**ASStm (Activation Signals for STM)**	0.95317	0.96102	0.95516	0.96491	0.9779	0.9788	1.0
**New Potential Deficit**	0.04683	0.03893	0.04484	0.03509	0.0221	0.0212	0.0

**Table 5:** This table comprises the classes that are used for training the intelligent agent.

Super Class	Class Title	No. of Images	Super Class	Class Title	No. of Images
**Person**	Human	400	**Sports**	Sports ball	200
**Vehicle**	Car	100	**Sports**	Tennis racket	100
**Vehicle**	Bus	50	**Kitchen**	Bottle	200
**Vehicle**	Train	50	**Kitchen**	Cup	200
**Animal**	Bird	100	**Kitchen**	Knife	100
**Animal**	Cat	200	**Food**	Apple	200
**Animal**	Dog	100	**Food**	Banana	200
**Animal**	Lion	100	**Food**	Sandwich	100
**Accessory**	Handbag	200	**Electronics**	Mobile	200
**Accessory**	Umbrella	100	**Furniture**	Chair	100

**Table 6:** This table defines the summary of experiments (attention). This table first defines attention type. Then in the goal object feature, it shows the queried object, and at last, it represents the status of attention towards an object.

Experiment No.	Attention Status on the Goal Object	Goal Object	Object/s Found	Goal Status
**1**	High	Null	Lion	-
**2**	High	Null	Cat	-
**3**	High	Null	Lion, cat	-
**4**	High	Cat	Cat	Yes
**5**	High	Dog	Dog	Yes
**6**	High	Dog	Dog	Yes
**7**	High	Car	Cars	Yes
**8**	Medium	Cup	Cup	Yes
**9**	Medium	Cup	Cup	Yes

**Table 7:** This table represents the summary of experiments (attention with emotions). This table defines how an agent performs and achieves its goal when it is influenced by emotional states.

Experiment No.	Attention Status on the Goal Object	Goal Object	Object/s Found	Previous Emotion State	Current Emotion State	Goal Status
**1**	High	Null	lion	Normal	Fear	-
**2**	High	Null	cat	Fear	Happy	-
**3**	Medium	Null	lion, cat	Happy	Fear	-
**4**	High	cat	cat, apple	Fear	Happy	Yes
**5**	High	Dog	Dog, cat	Fear	Fear	Yes
**6**	High	Dog	Dog, cat, apple, ball (miss perceived)	Fear	Fear	Yes
**7**	High	Car	Cars, ball (miss perceived)	Fear	Happy	Yes
**8**	Medium	Cup	Knives, cups, person	Happy	Fear	No
**9**	Medium	Cup	Knife, cup, persons	Fear	Fear	No

## Data Availability

The simulation files/data used to support the findings of this study are available from the corresponding author upon request.
